# Assessing the Impact of Early Learning Programs in Africa

**DOI:** 10.1002/cad.20224

**Published:** 2017-12-15

**Authors:** Amber Gove, Tracy Brunette, Jennae Bulat, Bidemi Carrol, Catherine Henny, Wykia Macon, Evangeline Nderu, Yasmin Sitabkhan

**Affiliations:** ^1^ RTI International; ^2^ Florida State University

## Abstract

We present results from early learning programs in six African countries: Ethiopia, Kenya, Liberia, Malawi, Tanzania, and Uganda. In partnership with ministries of education, RTI International has worked within government systems to support the design and deployment of locally contextualized materials, training, and assessment tools, with the goal of improving outcomes for early learners in primary schools, and in Kenya and Tanzania preprimary as well. Here we report on the experience and evidence of impact from specific programs in each country, including summary assessment results when available. In several countries with completed impact evaluations, there are significant and important learning gains of between 0.2 and 2.57 SD in effect size; in one case the percentage of students reaching grade‐level reading proficiency increased from 12% to 47%. In the context of increased urgency surrounding what UNESCO has called a “global learning crisis,” these experiences provide useful lessons for policymakers and practitioners alike.

## Introduction

Nearly 3 decades after the first of a series of global commitments to high‐quality education for all, many countries in Africa strive to keep their promise to ensure that all children learn. Rapid expansion of enrollments in the 1990s was not matched with concomitant increases in per‐pupil funding, preparation and hiring of teachers, or development and distribution of teaching and learning materials. In response to these challenges, many governments and their donor counterparts, including the U.S. Agency for International Development (USAID), increased efforts to improve learning outcomes. This paper presents an overview of the RTI International approach to learning improvement, followed by brief case studies on the implementation design and results of donor‐supported education programs in six African countries: Ethiopia, Kenya, Liberia, Malawi, Tanzania, and Uganda. In each case, RTI, a U.S.‐based nonprofit research institute, partnered with ministries of education to develop and implement research‐based education improvement programs tailored to each country context. Though each program design is localized to meet country‐ and region‐specific needs, the case studies indicate substantial overlap in the need for improvements in the quality and availability of teaching and learning materials, professional development and coaching for teachers, local language use in the classroom, better use of instructional time, and application of assessment results to monitor learning and inform implementation. The article concludes with a brief discussion section highlighting key lessons learned and a summary of the evidence of impact of the programs.

## Background

Few dispute the importance of learning to read within the first years of primary education, especially because learning that occurs early compounds over time. Students struggling to learn to read at the end of grade 1 must achieve twice the fluency gains in grade 2 to catch up with their peers (Good, Simmons, & Smith, 1998), and those who cannot quickly catch up tend to remain behind. Closing this deficit is even more challenging under the conditions prevalent in many low‐ and middle‐income countries (LMICs) in Africa.

Although most LMICs have achieved near‐universal primary enrollment, many face challenges in providing high‐quality learning opportunities. The United Nations Educational, Scientific, and Cultural Organization (UNESCO) estimates 250 million children (approximately 100 million in sub‐Saharan Africa) are not learning basic skills, a crisis with a global cost of $130 billion per year (UNESCO 2012, 2014). The elimination of school fees and subsequent enrollment increases have led to larger class sizes (UNESCO Institute for Statistics [UIS], 2006), teacher shortages and inadequate teacher preparation (Akyeampong, Pryor, Westbrook, & Lussier, 2011; Mulkeen, 2010), and limited or inefficient use of instructional time (Abadzi, 2009). Students often lack basic learning materials such as textbooks and other reading materials (Evans, 2010; RTI International, 2016).

In a decade of measuring and implementing early literacy programs, RTI has identified that, to be effective, programs must comprehensively address the “5 Ts” (Gove & Cvelich, 2011): effective *teaching*, access to high‐quality *texts* (materials), appropriate use of mother *tongue* languages, efficient use of instructional *time*, and use of formative and summative *tests* (assessments, including the Early Grade Reading Assessment [EGRA]) to inform instruction and monitor system improvement (Bulat et al., 2017). By training teachers to systematically introduce simple skills, then add complexities in a predetermined sequence, the model helps ensure all skills are covered. This approach combines explicit instruction in foundational skills with a range of holistic reading and writing experiences to foster appreciation of reading and writing—a balanced approach that is critical in contexts with sparse reading and writing opportunities. In our case studies, we demonstrate adaptation to individual country contexts.

## Country Case Studies

The following pages document how this model has been adapted to each country. Each case study includes a brief retrospective on the expansion of primary education followed by a summary of the intervention design in terms of project outputs (e.g., books printed, teachers trained) and results in terms of outcomes (e.g., evidence of improved classroom instruction or learning gains); when available, effect sizes are reported in Cohen's d. Because of word‐length constraints, this article provides only a surface introduction of the programs; for more detailed descriptions of the programs and their impact we refer the reader to specific project and evaluation reports, referenced in each case study and available as supplementary content in the online version of this article.

### Ethiopia

Ethiopia's change in government in 1991 and commitment to universal education sparked a reform focused on access, equity, efficiency, and quality (UNESCO, 2015). As Ethiopia set out to increase access, net primary enrollment rose steadily from 19% in 1994 to 86% in 2014. Quality suffered as the government struggled to provide sufficient human and physical resources (Ethiopia Ministry of Education, 2008). In 2010, an EGRA showed that by the end of grade 2, 34% of students were unable to correctly read one word and 48% of students scored a zero in comprehension (RTI International, 2010).

#### Intervention Design

In response to these challenges, USAID designed Reading for Ethiopia's Achievement Developed Technical Assistance (READ TA), one of four concurrent projects working at national scale focused on improving reading outcomes for 15 million students. READ TA's strategies included designing and developing reading and writing materials for classrooms and teacher training for grades 1–8; applying language‐specific teaching and learning methodologies; using technology and learning aids to support language learning; and providing technical assistance to support regional state education bureaus (RSEBs) and the national ministry in the training of teachers.

As READ TA approached the end of its fifth and final year (2017), the project had supported the RSEBs in developing more than 300 grade 1–8 student books and teachers’ guides, including materials for each of the seven selected mother tongues and English, in addition to readers for 12 other language communities. Over 2,500 teacher trainers delivered professional development to more than 170,000 teachers. The project team also worked with teachers to increase their knowledge of inclusive instructional methods and assistive technologies for students with vision and hearing impairments to facilitate identification and differentiation.

#### Results

As the program was delivered at national scale, without phasing to allow for a control group, an impact evaluation was not conducted. Nonetheless, to monitor progress and inform implementation, USAID conducted an EGRA in a subset of schools whose teachers had undergone training by the ministry; results showed that while students performed relatively well on the lower order listening comprehension subtask, about 70% obtained a zero on the more challenging reading comprehension subtask (American Institutes for Research, 2016). These results, coupled with select classroom observations conducted by READ TA, showed that although the new literacy materials may have been present in the classrooms, instructional practices had been more resistant to change.

### Kenya

In January 2003, the Kenyan government launched free primary education resulting in net enrollment rates rising from 62% in 2002 to 85% in 2012 (World Bank, 2017). The quality of education did not keep pace; a 2012 study found that only 3 in 10 grade 3 students were able to do grade 2 level work (Uwezo, 2012). In response to this and other evidence of low learning levels, the ministry, with financial support from USAID and the United Kingdom Department for International Development, designed and implemented the multiarmed cluster randomized controlled trial of the Primary Math and Reading (PRIMR) Initiative (2011–2014). Results from PRIMR indicated that well‐designed teachers’ guides and student workbooks, intensive teacher training, and targeted ongoing teacher support through coaches led to significant gains in student performance (Piper, Ralaingita, Akach, & King, 2016; Piper, Zuilkowski, & Mugenda, 2014).

#### Intervention Design

Building on the strength and success of PRIMR as well as the devolution of Early Childhood Development (ECD) to county governments in Kenya in 2013, the ministry initiated two projects to address the problem of quality in the classrooms. Both the *Tusome* (“Let's Read” in Kiswahili) Early Grade Reading Activity (2015–2019) as well as the *Tayari* (“Ready” in Kiswahili) Early Childhood Development Programme (2014–2018) were designed around existing government systems with a view to long‐term sustainability. By early 2017 *Tusome* had reached 22,300 public primary schools and 1,500 Alternative Provision of Basic Education and Training schools in major urban centers in all of Kenya's 47 counties. The program includes scaffolded teacher guides, student workbooks and leveled readers, and monthly coaching and assessment visits from government‐funded curriculum support officers.

To complement the primary program, *Tayari* is developing and testing a cost‐effective, scalable model of ECD that ensures preprimary‐aged children (4 and 5 years old) are mentally, physically, socially, and emotionally ready to start, and succeed, in primary school. *Tayari* is conducted as a cluster randomized controlled trial (groups of schools were randomly assigned to either a treatment or delayed‐entry control group) in collaboration with four county governments, with a view to scale nationally (if successful). Similar to *Tusome*, *Tayari* was incorporated into Kenya's existing systems—so as to increase the likelihood that the methods and materials could be replicated and scaled up. Student and teacher materials were developed in partnership with Kenya's Institute for Curriculum Development; student workbooks were distributed to all program schools at a ratio of one book per child. Teachers also receive training and monthly coaching visits from county government staff who themselves received training in how to support teacher practice in the classroom.

#### Results

First‐year evaluation results from *Tusome* and *Tayari* were promising. For *Tayari*, a longitudinal tracer study following 3,000 children was undertaken to better understand how school readiness skills transition over time. Researchers assessed children in January and again in October 2016, and compared them with children outside the program. *Tayari* children had much larger gains in learning than the control group: 18.4 points compared with 13.5 points on a School Readiness Index with an average effect size of 0.34 SD (Kwayumba, Piper, Oyanga, & Oyagi, 2017). Likewise, results from a recent external evaluation of *Tusome* revealed a reduction in grade 2 nonreaders in English from 38% to 12%, and an increase in pupils reading fluently from 12% to 47%. Over the same period, Kiswahili nonreaders in grade 2 declined from 43% to 19% whereas fluent readers increased from 4% to 12% (Management Sciences International, 2017). Though no counterfactual was possible because of *Tusome's* nationally scaled design, the effect size on targeted skills of between 0.4 and 2.57 SDs is promising and substantially larger than that documented by the PRIMR pilot. Expressed in terms of equivalent years of schooling, the gain in grade 2 learning is equal to an additional 1.3 years of school (Evans & Yuan, 2017).

### Liberia

Liberia has a turbulent history, from its origins as a colony for former African‐American slaves, to independence, to decades of civil war and a severe Ebola outbreak. With less than 2% of gross domestic product invested in education and net primary enrollment of around 40%, Liberia struggles to provide basic education services to its population, 43% of whom are under the age of 15 (Gove, Korda Poole, & Piper, 2016). In 2008, a World Bank‐funded EGRA revealed low reading levels, which prompted USAID to create the EGRA Plus program (2008–2010), designed to improve literacy instruction in the early primary grades. Evaluation results showed that students in the full treatment group made significant gains, including a 0.8 standard deviation (SD) effect in oral reading fluency (Piper & Korda, 2010). Building on this evidence, the 2010 Education Sector Plan identified several challenges, including lack of access to high‐quality data, high numbers of unqualified teachers, and lack of a system for professional development. In response, USAID's Liberia Teacher Training Program II (LTTP II) (2011–2015) was tasked with refining and then expanding the EGRA Plus program to focus on both literacy and mathematics in an additional two counties.

#### Intervention Design

The focus of LTTP II, led by FHI 360, was to strengthen teacher training and monitoring systems in coordination with the ministry; RTI was responsible for developing student and teacher materials and the instructional approach. A key activity was supporting teachers to enhance instruction in early literacy and mathematics. To do this, LTTP II created daily, scaffolded teacher guides that aligned with student workbooks. Teachers were trained on how to use the materials and given in‐class support by coaches who were trained to observe, give feedback, and model lessons for teachers.

#### Results

The implementation design allowed for a rigorous impact evaluation. Baseline, midterm, and endline data were collected for both Cohorts 1 and 2. LTTP II used a staggered entry cohort design, with randomization of school selection at the cluster level, allowing Cohort 2 to serve as a control group for Cohort 1 between baseline and midterm. Cohort 1 received intervention support between the baseline and midterm; Cohort 2 received support only between the midterm and endline data collection. However, because of challenges that arose because of the Ebola outbreak, Cohort 2 did not receive a full 2 years of implementation support.

Between baseline and midline, students in Cohort 1 improved from 4.8 to 14.2 correct words per minute on oral reading fluency, relative to almost no gains for Cohort 2 students, an effect size of 0.3 SD. Cohort 2 students improved their oral reading fluency between midterm and endline by almost five correct words per minute. Gains were not as large as Cohort 1, likely attributable to less instructional time and reduced teacher training and support because of the Ebola outbreak (King, Korda, Nordstrum, & Edwards, 2015). Endline results also indicated gains made by Cohort 1 were not sustained between midterm and endline. Although the Ebola outbreak may have had some impact, it also suggests that 2 years of the intervention, including classroom support and training, was insufficient to overcome the challenges that plague Liberia's education system. Expressed in terms of equivalent years of schooling, the gain in learning for Cohort 1 is equal to an additional 3.4 years of school (Evans & Yuan, 2017).

### Malawi

In 1994, the government launched free primary education; net primary enrollment increased from 58% to 97%in a 25‐year period, with more than half of that growth in the first year (Riddell, 2003; World Bank, 2017). As in other countries, enrollment growth was not matched by the provision of resources and learning did not keep pace. Malawi's performance on the 2007 Southern and Eastern Africa Consortium for Monitoring Educational Quality (SACMEQ III) assessment showed low average scores for grade 6 pupils and Malawi was the worst performer among the 15 countries that participated (Hungi et al., 2010).

A 2010 national EGRA confirmed these results: 72% of grade 2 and 42% of grade 4 students could not read a word of a simple story (RTI International, 2011). In response, USAID funded the Malawi Early Grade Reading Activity, which operated in 11 of 28 districts between 2013 and 2016. In 2014, the government developed the National Reading Strategy, a 5‐year plan for improving reading instruction in the early primary grades (Malawi Ministry of Education, Science and Technology, 2014). The strategy outlines an approach to supporting students in their early instruction in Chichewa, while transitioning to English from grade 5. USAID's Malawi Early Grade Reading Improvement Activity (MERIT) is the main vehicle for providing technical and financial support for implementing the strategy.

#### Intervention Design

MERIT is a 5‐year activity (2015–2020) whose goal is to support the ministry to improve the Chichewa and English reading skills of students in grades 1–4 nationwide through (a) improved reading instruction, (b) increased parental and community engagement in supporting reading, (c) safer learning environments, and (d) pathways for sustainability. The program rolled out to grade 1 in the 2016–17 school year and expanded to grades 2–4 in the 2017–18 school year. Over 20,000 teachers, head teachers, and educators were trained in the 2016–17 academic year using a three‐level cascade model. Each grade 1 teacher received 19 days of training on reading instruction, and school and region‐based staff also provided support to teachers through coaching. In addition, nearly 3 million books were distributed so that every public grade 1 pupil has her own book. MERIT also includes a social and behavior change communication campaign to encourage parents to read to their children after school and to ensure that children attend school regularly and on time.

#### Results

In November 2016, MERIT conducted a national baseline EGRA for pupils in grades 1 and 2. Owing to the simultaneous, nationally scaled implementation of the program a comparison group was not possible. Results indicated that students had weak reading skills across all subtasks and nearly all students (97% in grade 1 and 91% in grade 2) could not read any word of a simple story (LaTowsky & Pressley, 2017). Furthermore, less than 1% of students in either grade met the government benchmarks for reading. Factors related to low performance included large class sizes, high absenteeism, lack of teaching and learning materials, low capacity of teaching staff, and low system capacity to monitor and support learning. Class size in Malawi is particularly egregious: in 2015, the last year for which official data are available, the average grade 1 class had 153 children (UIS, 2017).

### Tanzania

Tanzania has made significant progress expanding access to primary education, with net enrollment increasing from 52% in 2000 to 80% in 2014 (UIS, 2017). Despite the improvement in access, results from a national EGRA revealed children were not mastering basic reading (RTI International, 2014). For example, only 8% of grade 2 students could read with comprehension (Marwa, 2014). In response, the government launched several initiatives to improve education policies and a “3Rs” program, aimed at improving reading, writing, and arithmetic instruction in preprimary through grade 2. Tanzania also adopted a preprimary policy in 2016, which called for 1 year of compulsory preprimary education for children ages 3 to 5.

#### Intervention Design

Tanzania's *Tusome Pamoja* Program (2016–2021), Kiswahili for “Let's Read Together,” builds on the understanding that establishing a strong foundation for lifelong learning and success begins in the earliest years. The program supports the government to strengthen the 3Rs performance of learners in grades 1–4 and to test preprimary interventions to boost the readiness skills of children entering grade 1. *Tusome Pamoja* aims to do this by (a) improving instruction through school‐based continuous professional development for teachers and the provision and use of quality instructional materials; (b) increasing parental and community engagement and support for student reading; and (c) improving the policy environment for reading by continuously collecting, storing, and using data at all levels. The program is implemented in four regions of Mainland Tanzania (Morogoro, Ruvuma, Iringa, Mtwara) and in Zanzibar.

As of mid‐2017, *Tusome Pamoja* had published 50 leveled and 12 decodable readers to be used in grades 1–2 and distributed more than 650,000 copies. The program was also in the process of printing 1.8 million decodable readers (three books for every grade 1 and 2 student), as part of a home reading strategy. *Tusome Pamoja* had also trained 13,000 primary and 315 preprimary teachers and was supporting creation of a school‐based continuous professional development framework. At the preprimary level, the program was reaching over 200 classrooms and had published a series of 24 storybooks and a curriculum implementation guide for teachers.

#### Results

In 2016, baseline student assessment data were collected at both the primary and preprimary levels. Similar to the findings of the 2013 national assessment (RTI International, 2014), baseline results uncovered very low reading, writing, math, and school readiness skills of children, with only 12% of grade 2 students meeting the oral reading fluency benchmark of 50 correct words per minute in Kiswahili (RTI International, 2017d). These results reinforce the overarching goals of the program, specifically the need for improved instruction and high‐quality materials development at all levels.

### Uganda

Since the late 1990s, Uganda's education system underwent a tremendous transformation, tripling the number of primary students to 8.6 million and increasing net enrollment to 92% (Uganda Ministry of Education and Sports, 2016). In 2007, the focus shifted to improving learning through a thematic curriculum and local language instruction starting in grade 1 (National Curriculum Development Centre, 2017). Yet poor outcomes persisted, as evidenced by low levels of reading achievement (Uganda National Examinations Board, 2016; Uwezo, 2016).

#### Intervention Design

The School Health and Reading Program (SHRP; 2012–2019) and the Literacy Achievement and Retention Activity (LARA; 2015–2020) are two early‐learning programs implemented by RTI, which also provides technical assistance to the Uganda Teacher and School Effectiveness Program (UTSEP; 2015–2019) implemented by the ministry with support from the Global Partnership for Education. The three programs, with a major (but not exclusive) focus on early grade reading, support 80% of public primary schools, reaching 6 million students in 9,750 schools. Through ministry systems, the programs support development of reading materials; methods and training for teaching reading; development of a literacy framework; incorporation of reading methods into the preservice teacher training curriculum; education finance reform; and periodic EGRAs. Outputs of the three programs as of mid‐2017 included development of 104 titles (teachers’ guides and pupil books) for grades 1–4 in 12 local languages and English, distribution of 5.18 million pupil books and teachers guides to schools, and training for 53,000 teachers.

#### Results

By late 2016, evidence emerged that teachers were changing their approach to teaching reading. Classroom observations found that 95% of learners were reading from printed material during the reading lesson in program schools compared to only 11% in control schools. Moreover, 43% of grade 2 teachers were guiding learners to make correct letter sounds, compared to no teachers in control schools (RTI International, 2017a). Program teachers were also more likely to prepare lesson plans and learners more likely to be reading from printed material and coming to school with pencils (RTI International, 2017c).

For the SHRP students who had 4 years of intervention, the effect sizes in oral reading fluency (correct words per minute) were Leblango, 0.28 standard deviations (SD); Runyunkore Rukiga, 0.39 SD; Ateso, 0.71 SD; and Luganda, 0.85 SD (RTI International, 2017b). Students were 1.5 to 6 times more likely to be reading 40 or more words per minute in these four languages in the program compared to control schools. For students who had 3 years of intervention, the effect sizes for oral reading fluency were Lugbarati 0.17, Acoli 0.95, Lumasaaba 0.95, and Runyoro Rutoro, 1.12 SD, and program learners were 1.5 to 9 times more likely to be reading 40 or more words per minute compared to control learners. At the end of grade 1, learners in LARA schools had also increased oral reading levels beyond increases found in SHRP control schools (RTI International, 2017c).

## Discussion

The experiences documented in the country case studies provide key lessons for policymakers and practitioners seeking to improve early learning results at medium to large scale. Table 1 provides a summary of each program in terms of students reached, as well as outcomes in terms of Cohen's d effect size (when available). As noted in the table, several programs are awaiting midterm review of results, and results were not available in time for this publication. Effect sizes range from 0.2 to 2.57, considered moderate to very large by most high‐income country program standards. Nonetheless, these gains need to be considered in the context of extremely low base‐level performance. If status quo learning is virtually nil, a small gain can translate into a large effect size.

**Table 1:** Summary of Highlighted Programs

Country/Program Name	Coverage	Grades	Languages	Students Targeted	Evaluation Design	Cohen's d Effect Size (SD)
Ethiopia/READ TA	National	1–8	7	15M	No control group	n/a
Kenya/*Tayari*	4 counties (out of 47)	K1, K2	2	75k	Cluster RCT	0.34
Kenya/*Tusome*	National	1–3	2	7M	No control group	0.4–2.57
Liberia/LTTP II	6 counties (out of 15)	1–3	1	450k	Cluster RCT	0.3
Malawi/MERIT	National	1–4	2	4.5M	No control group	n/a
Tanzania/*Tusome* Pamoja	5 regions (out of 26)	K1, K2, 1–3	1	1.4M	No control group	n/a
Uganda/SHRP	30 districts (out of 111)	1–4	12	1.5M	Cluster RCT	0.2–1.2
Uganda/LARA	28 districts (out of 111)	1–4	3	1.3M	Cluster RCT	n/a

*Note*: M = Millions, k = thousands, n/a = not available; Cluster randomized controlled trials (RCTs) with n/a results are those for which evaluation data are not available as of the press date of this publication.

Unfortunately, several programs lack a credible counterfactual, owing to the demand for simultaneous national or large‐scale rollout. Donors and host country governments should carefully weigh the political advantages of providing program benefits to everyone all at once relative to phased implementation that can result in improved management and increased fidelity of implementation. Lottery or randomized selection of groups of schools into phased entry treatment groups also allows for rigorous testing of programmatic effects. If national rollout is the only politically feasible approach, programs should include a well‐designed and evaluated trial phase to hone the intervention approach and determine effectiveness prior to rollout (as was the case in Kenya).

The key ingredients to fuel success, defined as achieving significant gains in learning outcomes, include professional development and support for *teachers* to improve their practice, the need for high‐quality teaching and learning materials (in particular student reading books/*texts*), working in a mother *tongue* or language that is familiar to the child, good use of instructional *time*, and regular assessment and *testing* to inform both classroom practice and program and system implementation. To varying degrees, these ingredients are present throughout these programs. The following pages provide a brief discussion of the issues and lessons that proved most salient under the programs reviewed in this paper.

### Teaching

Teacher professional development and support are critically important and extremely challenging to get right. Teachers vary widely in their level of expertise, belief systems, experience, and preparation, but we have found that nearly all teachers endeavor to do their best given the conditions they are in. One of the biggest challenges is overcoming low expectations of what students can achieve. Our task is to get teachers to just try the approach; we are confident that if they do, they will see improvements in reading outcomes. In each of the country programs we provided scaffolded lesson plans to guide classroom instruction and support teachers in their practice, along with student books. Teacher support programs ranged from 10 days of group professional development at a central site (Ethiopia) to monthly coaching visits from curriculum support officers (Kenya). Determining the best mix of ongoing professional development for teachers should be locally contextualized to each country, but we find that short (1‐ or 2‐day) group trainings followed by onsite monthly coaching and reinforcement have proven most effective in producing improved instructional practice and gains in learning outcomes.

### Text

Book production is one of the more complex aspects of these programs as it requires careful timing, planning, and the involvement of many kinds of expertise—from determining the scope and sequence of instruction to designing an effective book distribution system. Developing purposeful content and appropriately leveled activities and lesson plans that meet the instructional needs of students at all levels is just one part of the challenge. In many countries and languages, the combination of legal and infrastructure barriers means the market for textbooks and supplementary readers may not yet be of sufficient size or maturity for private publishers to justify the expense of production. In these cases of market failure, donors and governments step in to produce and print books for schools.

In the last decade, RTI has employed both external publishers and in‐house staff to design, produce, print, and deliver millions of books to schools. In all cases, having adequate time, usually at least a year, to develop and trial the materials and make revisions based on teacher feedback led to increased quality and improved outcomes. In the case of Kenya, Liberia, Malawi, and Uganda, precursor programs were used to develop and trial several iterations of materials. In Ethiopia, regional and language‐specific teams designed and developed materials for eight grades in seven languages in less than a year. In Tanzania, materials developed by the nongovernment organization Room to Read were ceded to the Tanzania Institute of Education for reprinting and use under the program. Copyright, including well‐intentioned promotion of Creative Commons licensing of materials developed with the support of donor funds, has proved to be a stumbling block for ministries accustomed to developing, licensing, and in some cases generating revenue from their own materials. Finally, creation of global quality standards and technical specifications for book production, akin to those created by the World Health Organization for pharmaceuticals, is urgently needed to improve the durability of teaching and learning materials in LMICs.

### Tongue

Country policies vary as to the duration of instruction in a given language, timing of transition to a second language, and teacher assignment policy and practice. Although African languages are increasingly used as a language of instruction, particularly in the early grades, most communities are linguistically heterogeneous, especially in urban areas. This means that even systems implementing an African language of instruction policy are not able to offer every child instruction in his or her home language. For example, although Kiswahili is now the official language of instruction throughout all cycles in Tanzania, 44.5% of students reported speaking one of the other 124 Tanzanian languages at home (RTI International, 2014).

The preceding cases show a range of approaches and similar variation in success. Although ministries of education need to decide what is best for their social and political context, language selection should facilitate, not hinder, learning. Students in these contexts already face many barriers; when feasible, we encourage countries to remove one more barrier by facilitating early reading instruction in a language the majority of students in a classroom speak and understand. To this end, we have developed guidance to inform policymakers and practitioners making challenging language policy decisions (RTI International, 2015), including suggestions for stakeholder engagement, materials development, and other practical steps for language use planning.

### Time

One of the minimum conditions for student learning is being in school and on task. Yet student and teacher absenteeism and limited time on task continue to plague many of the education systems highlighted here. There are many noneducation‐specific methods to improve attendance including electronic payments for teachers (eliminating the need to travel to an urban center to collect paychecks), improved transportation infrastructure to reduce travel times, and adequate sanitation facilities (which has particular benefits for girls). Regular unannounced coaching visits helped to reduce absenteeism in Kenya and Malawi, and the introduction of a safe schools initiative in Uganda has helped to reduce violence and increase attendance. Extremely large class sizes also affect instructional time as managing a classroom of 150 students (or more) is time consuming from a purely logistical standpoint, let alone one of delivering high‐quality instruction for all students. Time on task is a basic but necessary ingredient for improving learning; without sufficient attention to this foundational aspect, gains are nearly impossible to achieve and even the most well‐designed intervention will fail to meet expectations.

#### Test

Assessment (or testing) is comprised of both formative and summative assessment. Formative assessment is frequent and ongoing, designed to monitor student understanding of the skills and knowledge being taught and critically acting on that information to immediately adjust instruction. In each of the programs, teachers were trained in informal and formal assessment of student progress; e.g., in Malawi, teachers monitored student learning by asking students to show a thumb up or down to reflect their understanding of the task, whereas in Kenya, curriculum support officers conducted ongoing monitoring checks of student reading levels. Project teams also worked with local experts to develop summative EGRAs and in some cases Early Grade Mathematics Assessments (EGMA; Platas, Ketterlin‐Geller & Sitabkhan, 2016), which can be used to measure system and program effectiveness and identify gaps in instruction and areas for support. The use of assessment data at all levels of the projects has been of critical importance in these contexts, allowing programs to draw attention to equity gaps, inform the (re)design of materials, and track and monitor progress. In Uganda, project data identifying gaps in learning gains across language groups have highlighted the need for additional training and refinement of the program approach, whereas in Kenya, ministry officials rely on a dashboard system developed by the project to keep abreast of learning in schools.

Together, these key ingredients are contributing to important gains in learning outcomes in several countries. In others, there is still much work to be done. The increased reliance on assessment data to inform program design and effectiveness is particularly heartening: we are no longer going on faith that our well‐intended interventions are having an impact. Instead when we see evidence of the need for course correction we have the information to point us in the right direction. We hope the sharing of these experiences and lessons will prove helpful to others embarking on the important task of improving early learning for all children.
